# BM microenvironmental protection of CML cells from imatinib through Stat5/NF-κB signaling and reversal by Wogonin

**DOI:** 10.18632/oncotarget.8332

**Published:** 2016-03-24

**Authors:** Xuefen Xu, Xiaobo Zhang, Yicheng Liu, Lin Yang, Shaoliang Huang, Lu Lu, Shuhao Wang, Qinglong Guo, Li Zhao

**Affiliations:** ^1^ State Key Laboratory of Natural Medicines, Jiangsu Key Laboratory of Drug Design and Optimization, China Pharmaceutical University, Nanjing 210009, People's Republic of China; ^2^ Middle School of The City, Mei County, Baoji, Shaanxi 721000, People's Republic of China

**Keywords:** BM microenvironment, CD34+ subpopulation, Stat5, NF-κB, Wogonin

## Abstract

Constitutive Stat5 activation enhanced cell survival and resistance to imatinib (IM) in chronic myelogenous leukemia (CML) cells. However, the mechanism of Stat5 activation in mediating resistance to IM in bone marrow (BM) microenvironment has not been evaluated precisely. In this study, we reported HS-5-derived conditioned medium (CM) significantly enhanced IM resistance in K562 and KU812. Interestingly, upregulation of the proportion of CD34+ subpopulation was found in CML cells. Subsequently, the BCR/ABL-independent activation of Stat5 increased P-glycoprotein (P-gp) activity in CM-mediated protection of CML stem cells (LSCs) from IM. Further research revealed Stat5 activation increased the DNA binding activity of NF-κB though binding of p-Stat5 and p-RelA in nucleus. Moreover, highly acetylated RelA was required for Stat5-mediated RelA nuclear binding. The study further confirmed that Wogonin potentiated the inhibitory effects of IM on leukemia development by suppressing Stat5 pathway both in CM model and the K562 xenograft model. In summary, results clearly demonstrated BCR/ABL-independent Stat5 survival pathway could contribute to resistance of CML LSCs to IM in BM microenvironment and suggested that natural durgs effectively inhibiting Stat5 may be an attractive approach to overcome resistance to BCR/ABL kinase inhibitors.

## INTRODUCTION

IM has proven to be highly effective for inhibiting BCR-ABL tyrosine kinase inhibitors (TKI) in treatment of CML [1]. Unfortunately, cessation of drug treatment led to disease relapse in most CML patients [2]. To understand the mechanisms underlying CML persistence, previous studies have mainly focused on cell-intrinsic mechanisms such as BCR/ABL overexpression, Kinase mutations as well as drug influx and efflux regulations [3–5]. However, recent emerging evidence demonstrated that BM microenvironment provided critical signals to support LSCs preservation in development of drug resistance [6]. More importantly, there is increasing interest in inhibiting Stat5 directly to overcome resistance to BCR/ABL kinase inhibitors. But until now, the role of Stat5 signaling pathway in mediating resistance to TKI in the microenvironment has not been sufficient to elucidate.

BM microenvironment produces high local concentrations of paracrine- and autocrine- derived growth factors and cytokines, which protects LSCs from the effects of chemotherapy and small molecule-targeted therapies [7, 8]. CML cell lines with stromal cell-conditioned medium or fibronectin had been shown to contribute to resistance [9]. culture of primary CML CD34^+^ cells with CM from BM stroma attenuated the effect of TKI-induced apoptosis or inhibition of colony formation [10]. Although these results from cell lines and murine models indicated that microenvironmental interactions could protect CML cells from TKI-induced cell death, the underlying molecular mechanism of the BM microenvironment in conferring resistance was not well studied.

Stat5, one STAT family member, was found to be constitutively active in many forms of hematologic cancers, including CML [11]. Both JAK2 and BCR-ABL caused the activation of Stat5, which resulted in upregulated expression of genes driving cell cycle progression and promoting survival [12]. In BCR-ABL1-transformed cells, Stat5 activation depended on the ABL kinase activity because IM completely abrogated phosphorylation of Stat5 [13]. constitutively activated Stat5 has been proved to be independent of JAK2 in BCR-ABL1 cells [14]. However, reports had also shown a correlation between elevated Stat5 phosphorylation and IM resistance [15]. High levels of Stat5 attenuated the inhibitory effects of IM on BCR-ABL1 cells. Specific targeting of Stat5 activity increased eradication of BCR-ABL1 cells, even in IM-resistant cells [17, 16, 17].

BM microenvironment can protect CML-LSCs from TKI-induced cell death or inhibition of colony formation, indicating that other survival pathways were activated. Moreover, these survival pathways were identified as BCR-ABL kinase- independent mechanisms. For instance, JAK2 was important among signaling pathways induced by hematopoietic growth factors such as granulocyte macrophage colony-stimulating factor (GM-CSF) and interleukin (IL)-3 [18]. Compelling evidence was supported by IL-3 and G-CSF as mediators of autocrine growth loops in primary human CML CD34^+^ cells to activate Stat5 [19]. Thus, cytokine- activated JAK2/Stat5 signaling might indeed be dispensable when BCR-ABL was fully active, but became critical when BCR-ABL was inhibited, which had also been identified as other potential targets.

Meanwhile, another transcription factor NF-κB could be activated by several growth factors, including GM-CSF and IL-3 in hematopoietic cells [20, 21]. Intriguingly, blockade of JAK3 activity diminished the binding of Stat5 to BCL10-SBR in MT-2 cells, which decreased NF-κB activity and BCL10 protein expression. Moreover, specific Stat5 depletion was correlated with decreased NF-κB DNA-binding, cell viability in both the presence and absence of IL-2 [22]. In addition, NF-κB-dependent gene expression involved a growing number of coactivators and Stat5-dependent signals might regulate the function of such transcriptional coactivators [23]. In this regard, it would be essential to identify the specific mechanism for better understanding of cross-talk between the Stat5 and NF-κB pathways.

It is highly possible that Stat5 reactivation in BCR-ABL- independent mechanisms may confer to CML LSCs preservation in BM microenvironment with treatment of IM. To further clarify this hypothesis, our study was performed in established CM model and in animal model. In this report, data showed that CM significantly inhibited apoptosis and enhanced IM resistance in CML cells. To our surprise, the proportion of CD34^+^ cells in CML cells was increased by treatment with IM in CM. In this context, we evaluated the role of Stat5 signaling pathway in modulating response to TKI treatment. To further comfirm this model and its related mechanism, one of traditional chinese medicine with lowtoxicity and general safety was used. Wogonin (5, 7-dihydroxy-8- methoxyflavone), the major flavonoids isolated from the root of Scutellaria baicalensis Georgi, is the most promising anticancer candidate [24] due to its antiproliferating [25], cell migration-inhibiting [26, 27], angiogenesis-inhibiting [28, 29] and differentiation- inducing activities [30]. Wogonin enhanced etoposide-induced apoptosis by inhibiting P-glycoprotein [31]. Oroxylin A, another active flavonoids sensitized multi-drug resistance of human hepatoma BEL7402/5-FU cells to 5-FU via downregulation of P-glycoprotein expression [32]. Recently, Yan Zhong [33] reported that Wogonin effectively reversed multi-drug resistance of MCF-7/DOX cells by inhibiting Nrf2 signaling. In the paper, results indicated that Wogonin potentiated the inhibitory effect of IM on CML cells in BM microenvironment, and may be an attractive drug by targeting Stat5 directly to overcome resistance to BCR/ABL kinase inhibitors.

## RESULTS

### CM protected CML cells from IM-induced cell death

**Figure 1:**
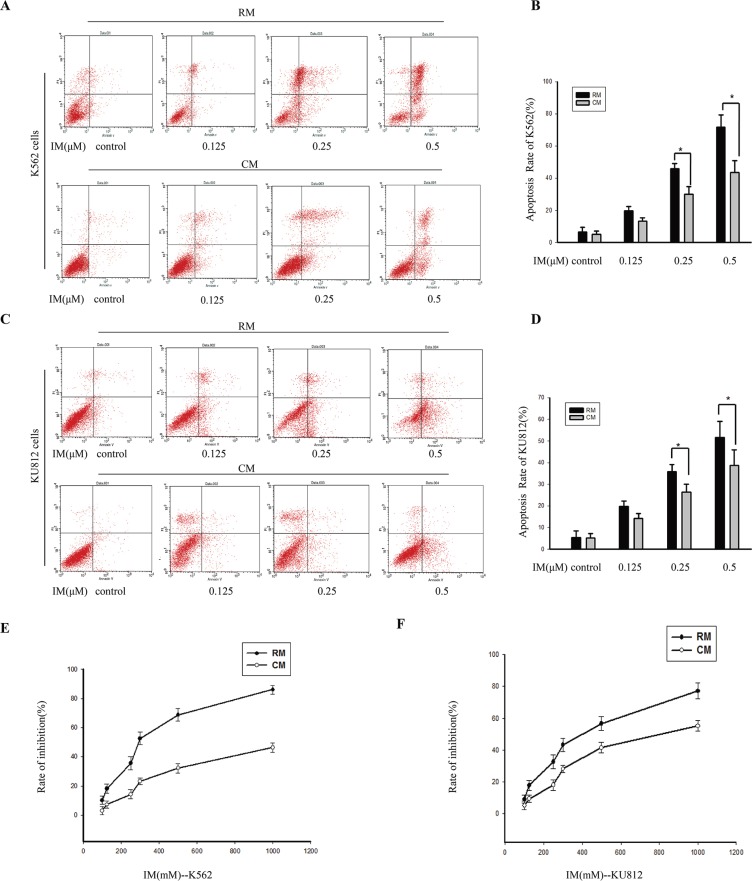
The effects of CM on attenuating IM-induced apoptosis in K562 cells and KU812 cells K562 cells and KU812 cells were cultured in RM or CM for 12 h and then treated with various concentrations of IM or 0.1% DMSO for 36 h, respectively. (**A**) and (**C**) Apoptosis was measured by Annexin V-PI double staining assay after treatment with IM in RM or CM. (**B**) and (**D**) The apoptotic rates were analyzed by flow cytometry. *p < 0.05 compared with RM. (**E**) The growth inhibition effect of IM on K562 cells in RM and CM. (**F**) The growth inhibition effect of IM on KU812 cells in RM and CM, and the inhibition rate (%) was calculated. Data were expressed as means ± SD of three independent experiments.

To establish bone marrow stroma-derived soluble factors in conferring IM resistance on CML, we used immortalized human bone marrow stromal cells as a model of the microenvironment [34]. Results showed that CM from HS-5 protected CML cells from IM-induced cell death. K562 cells and KU812 cells were incubated in RM or CM for 12 h and then exposed to different concentrations of IM for 36 h. As shown in Figure 1A and 1C, cells were labeled with Annexin V-PI and analyzed for apoptosis by flow cytometry (Figure 1B and 1D). Compared with RM, the IM inducing-apoptosis of K562 cells in CM was reduced by 6.51% ± 1.04, 15.81% ± 3.15 and 30.61 ± 5.47 at concentrations of 0.125, 0.25 and 0.5 μM, respectively. Similarly, in KU812 cells, culture with CM reduced the IM inducing-apoptosis by 5.51% ± 1.21, 9.45% ± 2.02 and 13.64% ± 2.42 at concentrations of 0.125, 0.25 and 0.5 μM, respectively. We next analyzed the effect of CM on the viability of CML cells by MTT. Compared with RM, CM significantly overcame IM-induced growth inhibition of K562 cells even in the presence of a high drug concentration (Figure 1E). Similar results were also found in KU812 cells (Figure 1F). Although culture with CM significantly changed inhibition rate of two cell types treated with IM, but the degree of change in K562 cells was significantly greater than in KU812 cells, both with IM treatment.

**Figure 2:**
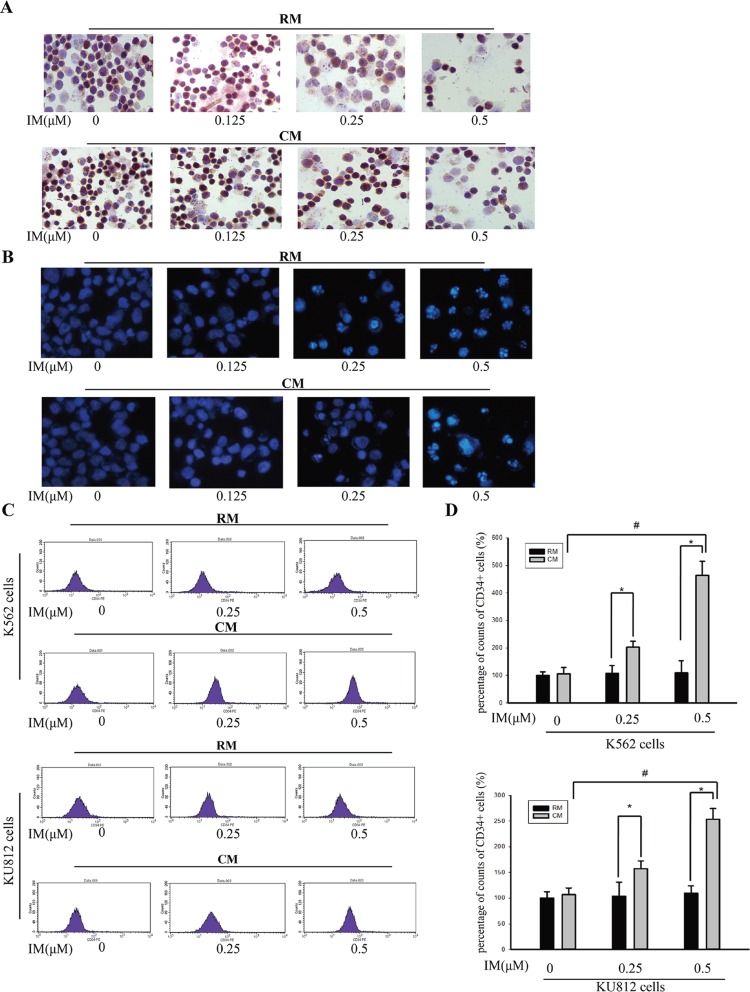
CM protected K562 cells and KU812 cells from IM-induced apoptosis K562 cells and KU812 cells were cultured in RM or CM for 12 h and then treated with various concentrations of IM or 0.1% DMSO for 36 h, respectively. (**A**) Cell proliferation of K562 cells was detected by Ki67 expression. (**B**) Apoptotic cells were observed by DAPI staining. (**C**) CD34^+^ subpopulation in K562 cells or KU812 cells was detected by flow cytometry. (**D**) The percentage of CD34^+^ cells in K562 or KU812 was analyzed by flow cytometry. All results were represented as the mean ± SD of three independent experiments. *p < 0.05 compared with RM, ^#^*p* < 0.05 compared with untreated K562 cells or KU812 cells in CM.

To further address the contribution of soluble factors in mediating the proliferation of K562 cells, we performed Ki67 cell proliferation assay. CM significantly increased the Ki67 indexes of K562 cells treated with IM (Figure 2A). DAPI stained nuclei showed bright condensed dots indicative of apoptotic bodies and significant alterations of the nucleus. As illustrated in Figure 2B, antipoptosis phenomenon was exhibited more markedly in K562 cells in CM with IM treatment. In this setting, we determined whether CM-mediated protection in CML cells was associated with CML LSCs. Specifically, culture with CM significantly increased the proportion of CD34^+^ cells with IM treatment while no change without IM treatment (Figure 2C and 2D). By flow cytometry assay, we observed exposure with 0.5 μM IM selected for cells expressing CD34. These results suggested that CM not only protected CML cells from apoptosis but also enhanced maintenance of CML stem cells during IM treatment. Thus, this might contribute to the failure of BCR-ABL inhibitors to eradicate minimal residual disease.

### Stat5 and P-gp contributed to resistance toward IM in CM

**Figure 3:**
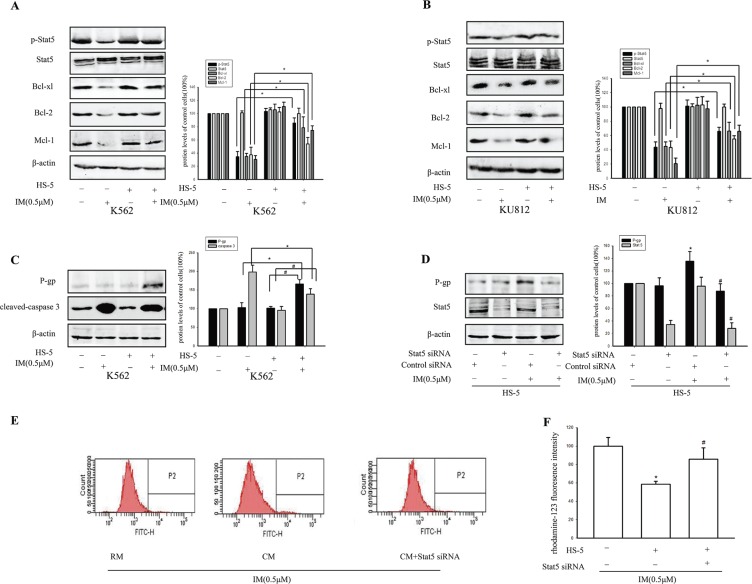
Activation of stat5 and P-gp contributed to resistance toward IM in CM K562 cells and KU812 cells were cultured in RM or CM for 12 h and then treated with 0.5 μM IM or 0.1% DMSO for 36 h. (**A** and **B**) The expressions of Stat5, p-Stat5, Bcl-xl, Bcl-2 and Mcl-1 was determined using Western blot analysis in K562 cells or KU812 cells, respectively. *p < 0.05 versus treatment with 0.5 μM IM in RM. (**C**) The expressions of P-gp and cleaved-caspase3 was determined by Western blot in K562 cells. *p < 0.05 compared with treatment with 0.5 μM IM in RM, ^#^*p* < 0.05 compared with untreated K562 cells in CM. (**D**) K562 cells were transfected with Stat5 siRNA or control siRNA, then treated with or without 0.5 μM IM for 36 h in CM. The expression of P-gp and Stat5 was analyzed by Western blot. *p < 0.05 versus K562 cells transfected with control siRNA. ^#^*p* < 0.05 compared with control siRNA treated with IM. (**E**) Intracellular rhodamine-123 fluorescence was measured to assess the efflux pump function of P-gp in CM model cells. The K562 cells were transfected with or without Stat5 siRNA, then exposed to 0.5 μM IM in RM or CM for 36 h. (**F**) The fluorescence intensity was measured by flow cytometry. *p < 0.05 compared with RM, ^#^*p* < 0.05 compared with untransfected K562 cells treated with IM in CM. Each value represented the mean ± SD of three independent experiments.

To further study the mechanism of maintenance of CML stem cells in CM, protein levels were detected by western blot. Many of the growth factors and cytokines were reported to activate members of the JAK family, and, subsequently, Stat5 [28]. As shown in Figure 3A and 3B, increased p-Stat5 was observed in CM compared with RM in K562 and KU812 cells, both with IM treatment, but no significantly change was observed without IM treatment. The different change with or without IM might be due to the very low proportion of CD34^+^ in CML cells (0.99–2.07%), which was shown at G0 phase almost. Then, the expression of p-Stat5 in the CML stem cells after IM treatment was activated in BM microenvironment. Furthermore, culture with CM significantly enhanced the expression of Stat5-target genes including Mcl-1, Bcl-xl and Bcl-2 after IM treatment. Meanwhile, similar increased results were also obtained in KU812 cells in the presence of IM. According to the increased degree of p-Stat5, we chose K562 cells for next study.

**Figure 4:**
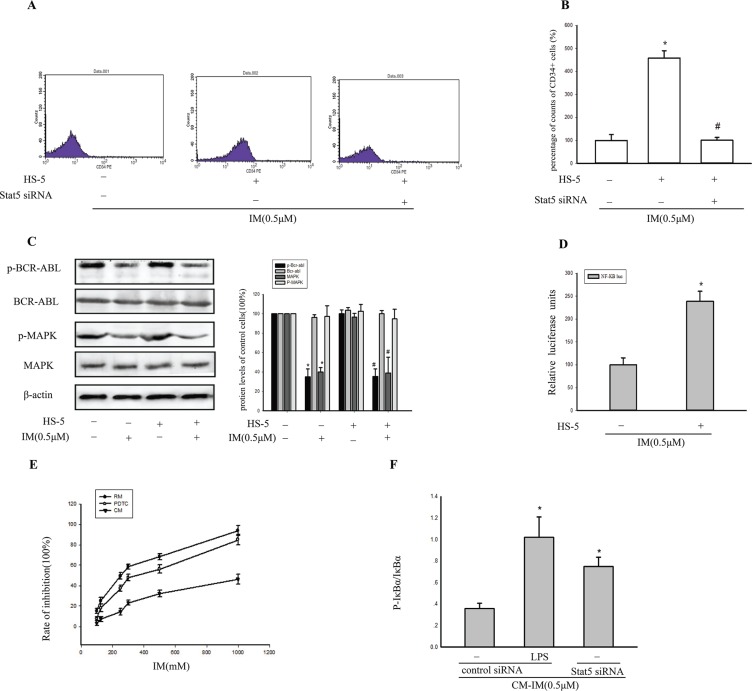
BCR/ABL-independent activation of stat5 maintained NF-κB activity (**A**) After transfection with Stat5 siRNA, CD34+ subpopulation in K562 cells was detected by flow cytometry. (**B**) The percentage of CD34+ cells in K562 was analyzed by flow cytometry. (**C**) The level of BCR-ABL, p-BCR-ABL, MAPK and p-MAPK was detected by Western blot analysis in K562 cells. *p < 0.05 compared with RM, ^#^*p* < 0.05 compared with untreated K562 cells in CM. (**D**) Cells were cotransfected with pNFκB-TA-luc with Renilla luciferase reporter (as internal control) for 24 h and then treated with IM for 36 h in RM or in CM. *p < 0.05 versus treatment with 0.5 μM IM in RM. (**E**) 4 h after treatment with 20 μM PDTC in CM, viability of K562 cells was determined by MTT assay after treatment with various concentrations of IM in RM or CM, and the inhibition rate (%) was calculated. (**F**) Cells were pretreated with 1 μg/ml LPS for 4 h, then transfected with control siRNA or Stat5 siRNA in CM. The ratio of p-IkBα/IkBα was analyzed by Western blot. *p < 0.05 versus control siRNA group. All results were represented as the mean ± SD of three independent experiments.

Subsequently, when K562 cells were treated with 0.5 μM IM in CM, pro-apoptotic protein caspase 3 was significantly reduced compared with RM. However, the expression of P-gp, a well-characterized drug transporter, was increased by 62.12% ± 11.34%. In contrast, there was no significant change in P-gp with or without supernatant of HS-5 in the absence of IM. Additionally, the degree of intracellular accumulation of rhodamine-123, which is a well-established P-gp substrate, was gauged by its fluorescence intensity. As shown in Figure 3E and 3F, compared with RM after treatment with IM for 36 h, intracellular accumulation of rhodamine-123 was reduced to 0.63 fold which suggested tumor microenvironment increased P-gp activity. Thus, we postulated that, BM microenvironment enhanced maintenance of CML stem cells by activating Stat5, consequently resulted in upregulation of P-gp activity. In support of this hypothesis, Stat5 was knockdown to further clarify the relationship of Stat5 and P-gp in K562 cells in CM. As Figure 3D indicated, P-gp was also reduced by Stat5siRNA in K562 cells in CM following treatment with IM, but no significant difference in RM. This difference suggested that the activation of Stat5 signaling pathway by CM was distinct from RM. Moreover, transfection with siRNA targeting Stat5 had a significantly reversal effect on intracellular accumulation of rhodamine-123 in CM model cells with 0.5 μM IM treatment (Figure 3E and 3F). In addition, significantly, the increase of the proportion of CD34^+^ cells in CM was also reversed by transfection with siRNA (Figure 4A and 4B). Therefore, the up-regulating expression of P-gp and the increased P-gp activity may be related with activation of Stat5 in the tumor microenvironment.

### BCR/ABL-independent activation of Stat5 maintained tumor NF-κB activity

Stat5 is the key substrate of BCR/ABL and JAKs. Next, we asked whether CM-induced Stat5 activation in K562 cells was dependent on BCR/ABL. BCR/ABL kinase activation status was assessed using western blotting for detection of p-BCR/ABL and p-MAPK. Neither RM nor CM interfered with the inhibition of p-BCR/ABL and p-MAPK by IM (Figure 4C). In fact, IM completely dephosphorylated BCR/ABL, indicating that CM-mediated IM resistance was BCR/ABL independent. In contrast, p-Stat5 was exclusively inhibited in the presence of RM, but not CM (Figure 3A). Thus, CM-induced IM resistance was associated with BCR/ABL- independent activation of Stat5.

We further demonstrated that CM-induced IM resistance cells with activation of Stat5 showed constitutive NF-κB activity by luciferase assays (Figure 4D). K562 cells were more sensitive to IM when pretreated with the specific NF-κB inhibitor PDTC for 4 h and then exposed to IM for 36 h in CM (Figure 4E). Those findings provided a supposition for a functional interaction between Stat5 and NF-κB in CM-induced IM resistance cells. To verify the possibility, we first investigated the p-IκBα/IκBα ratio with or without Stat5 in K562 cells in CM. Data showed that the ratio of p-IκBα to IκBα was higher in Stat5-knockdown cells, which indicated Stat5 signaling negatively regulated IKK activity (Figure 4F). However, these findings raised the question of how activated Stat5 and NF-κB could coexist in CM-induced IM resistance cells.

**Figure 5:**
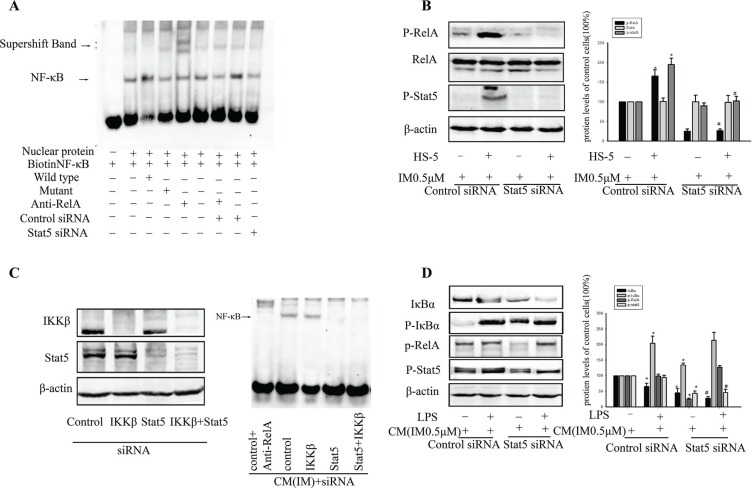
Stat5- activated NF-κB depended on RelA phosphorylation (**A**) K562 cells were transfected with Stat5 siRNA or control siRNA, then treated with or without 0.5 μM IM for 36 h in CM. NF-κB DNA binding activity was assessed by EMSA. (**B**) Transfection in K562 cells was treated with 0.5 μM IM in RM or CM. The level of RelA, p-RelA and p-Stat5 was detected by Western blot. *p < 0.05 compared with control siRNA in RM, ^#^*p* < 0.05 compared with control siRNA in CM. (**C**) Stat5 was required for maintaining RelA activity while less dependent of IKKβ activation in CM-induced IM resistance cells. Cells were transfected with the indicated siRNAs, and NF-κB DNA binding activity was assessed by EMSA. (**D**) Cells were pretreated with or without 1 μg/ml LPS for 4 h, then transfected with the indicated siRNAs. The level of IκBα, p-IκBα, p-RelA and p-Stat5 was determined by Western blot analysis in K562 cells. *p < 0.05 compared with control siRNA in CM, ^#^*p* < 0.05 compared with control siRNA added with LPS in CM. All results were represented as the mean ± SD of three independent experiments.

To further ascertain the interaction between Stat5 and NF-κB, we examined NF-κB activity by EMSA. As illustrated in Figure 5A, data showed p-RelA was constitutively bound to its consensus DNA sequence. When Stat5 was knocked down by siRNA, the binding of NF-κB activity was reduced in CM-induced IM resistance cells (Figure 5A). Meanwhile, we also observed increased p-RelA and p-Stat5 in resistant K562 cells (Figure 5B). There was a greater reduction of RelA activity in K562 cells when Stat5 activity was abrogated by Stat5siRNA (Figure 5B). It was likely due to the interruption of Stat5- mediated crosstalk between CML stem cells and the tumor microenvironment. Given above findings suggested a possible requirement of Stat5 for constitutive RelA activity in CM-induced IM resistance CD34^+^ cells. These observations were opposite of what the primary effect of Stat5 on the NF-κB pathway in CD34^+^ cells was inhibition of IKK. Next, we investigated whether IKK were responsible for maintaining constitutive NF-κB activity. In contrast to Stat5siRNA treatment, silencing IKKβ alone did not affect nuclear NF-κB activity over a 36 h period (Figure 5C). However, silencing both Stat5 and IKKβ significantly reduced NF-κB activity in cancer cells. These findings indicated that maintenance of existing constitutive NF-κB activity in resistance CML stem cells depended more on Stat5 than IKK activity, but they did not exclude the need for IKK to initiate NF-κB activation by facilitating its nuclear translocation.

Having shown that Stat5 also inhibited IKK activity, we next analyzed whether Stat5-activated NF-κB depended on NF-κB activity being initiated by a proinflammatory stimulus. As shown in Figure 5D, both the proinflammatory factor LPS and HS-5 supernatant were able to stimulate RelA activity in K562 cells in IM, as indicated by an increase in p-RelA. However, while LPS-induced p-RelA was associated with upregulation of p-IκBα, CM-induced p-RelA was associated with Stat5 activation but not with an increase in p-IκBα. Knockdown of Stat5 resulted in upregulation of LPS-induced RelA activity due to abrogation of IKKβ inhibition. These data demonstrated that the mechanism of the effects of Stat5 on RelA activity was distinct from that of proinflammatory stimulus. Taken together, BCR/ABL-independent activation of Stat5 maintained constitutive NF-κB activity, which might also contribute to CM-induced IM resistance.

### Stat5 was required for p-RelA in nuclear

**Figure 6:**
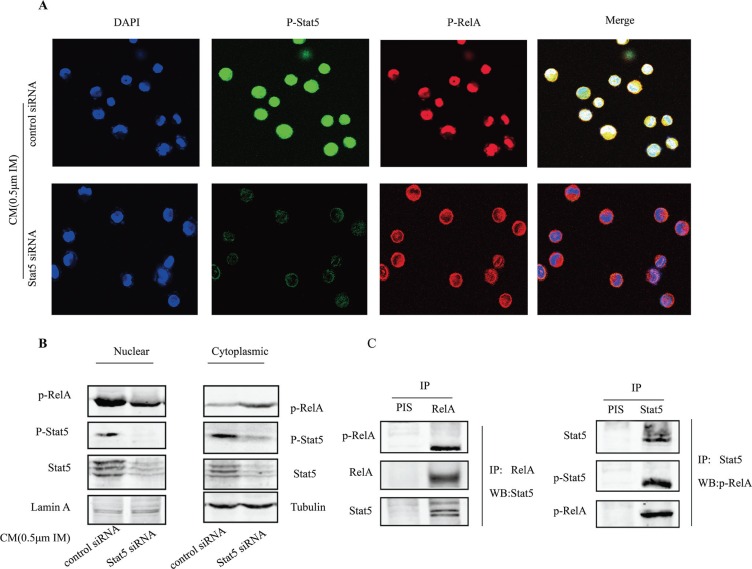
Stat5 was required for p-RelA in nuclear (**A**) Nuclear translocalization of p-Stat5 (green) and p-RelA (red) was observed by confocal microscopy after immunofluorescence staining of K562 cells treated with Stat5 or control siRNAs. DAPI was used to stain nuclei. (**B**) K562 cells were transfected with Stat5 siRNA or control siRNA, then treated with 0.5 μM IM for 36 h in CM. The expression of nuclear and cytoplasmic protein was extracted and assessed by Western blot in treated K562 cells. (**C**) Nuclear extracts from K562 cells after treatment were immunoprecipitated using anti-Stat5 antibody or anti-RelA or preimmune serum (PIS) control, followed by Western blot with indicated antibodies. All results represented the mean ± SD of three independent experiments.

To further explore how Stat5 might enhance RelA activity in CM-induced IM resistance cells, we utilized immunofluorescence staining and confocal microscopy to observe p-Stat5 and p-RelA. Our results showed that silencing Stat5 by siRNA resulted in significant reduction of p-RelA levels in the nucleus (Figure 6A). To confirm this, nuclear and cytoplasmic extracts were prepared from resistance cells, and levels of phospho- and total Stat5 and RelA proteins were examined by western blot. Consistent with the results in Figure 6A, knocking down Stat5 decreased nuclear p-RelA levels, whereas the cytoplasmic protein p-RelA was increased (Figure 6B). Additionally, IP assay with either anti-RelA or anti-Stat3 antibody was utilized to examine the binding of p-Stat5 and p-RelA. The data showed that p-Stat5 and p-RelA also physically bound with each other in nucleus (Figure 6C).

### Stat5 maintained constitutive NF-κB activity via RelA acetylation

**Figure 7:**
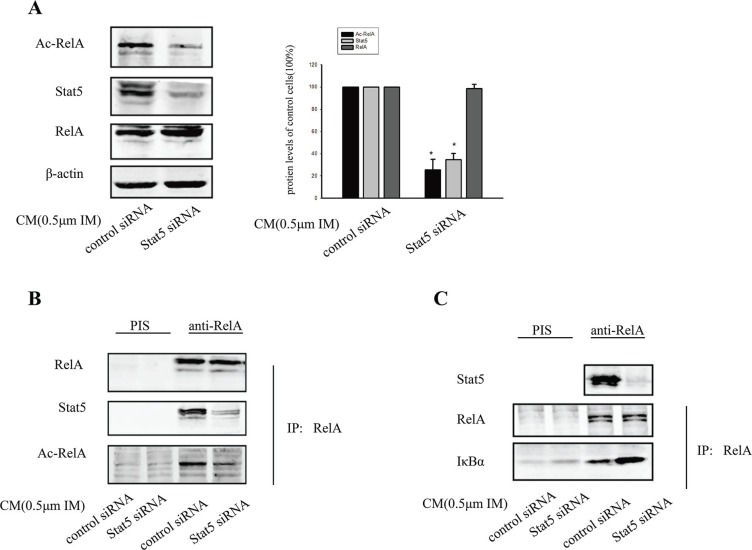
RelA acetylation was critical for Stat5 maintained constitutive NF-kB activity (**A**) Knockdown of Stat5 decreased RelA acetylation in CM-induced K562 cells treated with 0.5 μM IM. K562 cells were transfected with Stat5 siRNA or control siRNA. *p < 0.05 compared with control siRNA in CM. (**B**) Knockdown of Stat5 decreased interaction of Stat5 with Ac-RelA. siRNA-transfected cells were immunoprecipitated with anti-RelA antibody or preimmune serum (PIS) control, followed by Western blot with indicated antibodies. (**C**) Stat5 activity inhibited nuclear RelA's affinity to IκBα in CM-induced IM resistance K562 cells. Nuclear RelA from K562 cells treated with Stat5 siRNA was allowed to interact with IκBα, followed by Western blot analysis. The effects of Stat5 siRNA are shown. Results represented the mean ± SD of three independent experiments.

Here, we wanted to further clarify the potential mechanism that could facilitate Stat5-induced p-RelA nuclear accumulation in BCR/ABL-independent IM resistance. Previous studies have shown that RelA acetylation could contribute to its nuclear retention [29]. Importantly, RelA phosphorylation was required for RelA acetylation. Thus, we also considered the possibility that BCR/ABL-independent activation of Stat5 might induce RelA acetylation, thereby prolonging nuclear accumulation of p-RelA in CM-induced IM resistance cells. When Stat5 was knocked down by siRNA, the expression of acetylated RelA (Ac-RelA) was effectively blocked, but total levels of endogenous RelA protein was not affected (Figure 7A). Additionally, nuclear extracts were prepared from treated K562 cells for IP assay. Data showed that the binding of Stat5 and Ac-RelA was also significantly diminished by transfecting with Stat5 siRNA (Figure 7B). These findings prompted us to further investigate a potential role of Stat5 in regulating RelA acetylation. Nuclear extracts were immunoprecipitated by incubation with anti-RelA antibody, and RelA complexes were pulled down by streptavidin-conjugated magnetic beads. We found that nuclear RelA from Stat5 knockdown tumor cells showed increased interaction with IκBα. The findings suggested that Stat5 activity inhibited RelA affinity for IκBα (Figure 7C), which might contribute to interact with p-Stat5 and Ac-RelA.

### Wogonin overcame IM resistance of K562 cells within CM model

Aforementioned, it clearly showed that the enhanced viability of CD34^+^ stem cells played an important role in CM-induced IM resistance through activation of BCR/ABL-independent Stat5 signaling. The data also provided preclinical rationale for using Stat5-inhibitors to increase the efficacy of IM. Here, the inhibition effect of Wogonin on Stat5 signaling pathway was investigated in K562 cells in CM.

**Figure 8:**
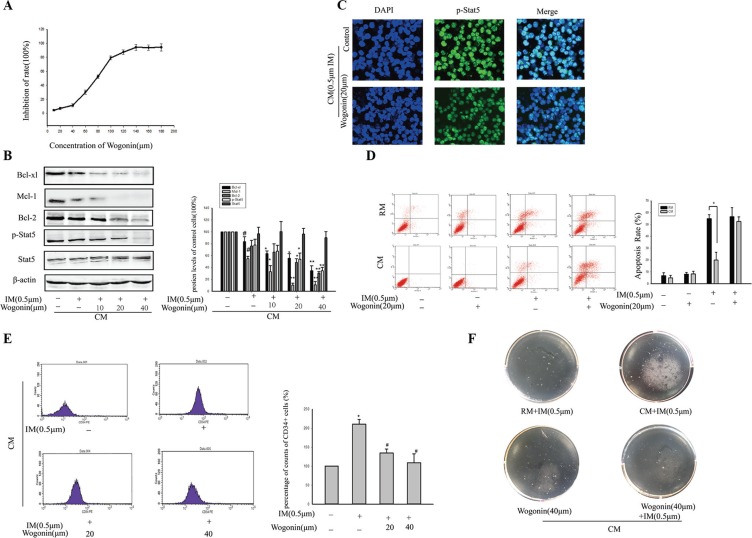
Wogonin overcame IM resistance of K562 cells within BM microenvironment model (**A**) Effects of Wogonin on viability of K562 cells were determined by MTT assay. The cells were treated with various concentrations of Wogonin for 36 h in CM. (**B**) K562 cells were exposed to 0.5 μM IM in the absence or presence of different concentrations of Wogonin for 36 h in CM. Stat5 signaling pathway was assessed by Western blot analysis in treated K562 cells. *p < 0.05 versus treatment with 0.5 μM IM in CM. ^#^*p* < 0.05 compared with untreated K562 cells. (**C**) Nuclear translocalization of p-Stat5 (green) was observed by confocal microscopy after immunofluorescence staining of K562 cells treated with or without 20 μM Wogonin in 0.5 μM IM-induced CM. (**D**) Apoptosis of K562 cells was measured by Annexin V-PI double staining assay after treatment with IM or Wogonin or the combination in RM or CM. The apoptotic rates of K562 cells were analyzed by flow cytometry. *p < 0.05 compared with RM. (**E**) K562 cells were exposed to 0.5 μM IM in the absence or presence of different concentrations of Wogonin for 36 h. CD34+ subpopulation in K562 cells was detected by flow cytometry. *p < 0.05 compared with CM. ^#^*p* < 0.05 versus treatment with 0.5 μM IM in CM. (**F**) Soft-sugar-colony forming experiment was performed to ascertain Wogonin reversal effect. Data was represented as means ± SD of three independent experiments.

MTT method was used to test the toxicity of Wogonin on K562 cells in CM. The viability of K562 cells was inhibited by Wogonin in a dose-dependent manner (Figure 8A). To minimize the effect of Wogonin itself on K562 cells growth, we chose lower concentrations (10, 20 and 40 μM) of Wogonin in the reversal experiments. After combination treatment with Wogonin and IM in K562 cells in CM for 36 h, the Stat5 protein level was detected by western blot. Figure 8B showed that p-Stat5 was greatly decreased by the combination treatment in CM. But the level of total Stat5 was not affected by the combination of Wogonin and IM. Immunofluorescence confirmed more p-Stat5 was translocated from the cytoplasm to the nucleus in HS-5 supernatant and Wogonin could reverse this process (Figure 8C). Additionally, Wogonin significantly decreased the expression of Stat5-target genes including Mcl-1, Bcl-2, Bcl-xl in CM-induced IM resistance cells (Figure 8B). These data indicated Wogonin could inhibit Stat5 signaling pathway in K562 cells within microenvironment model. So the synergistic effects of Wogonin and IM on model cells were investigated. K562 cells were incubated in HS-5 supernatant for 12 h, and then exposed to Wogonin and IM for 36 h. The combination of two drugs resulted in a significantly increased apoptosis rate of K562 cells (Figure 8D). Meanwhile, the proportion of CD34^+^ cells from K562 cells was also diminished in the presence of CM (Figure 8E). Furthermore, colony-forming capacity of CML CD34^+^ stem cells in CM was decreased in combined group compared with IM single group (Figure 8F). Taken together, within CM model, the weakly-toxic concentration of Wogonin could reverse K562 cells resistance to IM through inhibiting viability of CD34^+^ stem cells via Stat5 inactivation.

### Wogonin potentiated the inhibitory effect of IM on leukemia development *in vivo*

**Figure 9:**
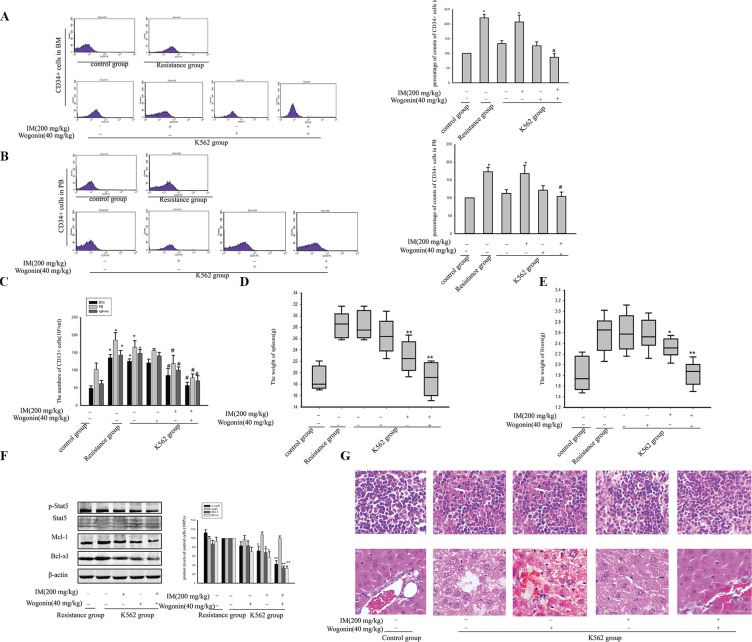
Wogonin potentiated the inhibitory effect of IM on leukemia development in NOD/SCID mice Tumor xenografts inoculated with K562 cells cultured with CM (K562 group) and CM-induced IM resistance cells (resistance group). Then the animals transplanted K562 cells were treated with IM (200 mg/kg) daily orally; Wogonin (40 mg/kg) intravenous injection were performed every other day for 4 weeks. (**A**) CD34^+^ cells of BM were detected by flow cytometry. *p < 0.05 compared with control group. ^#^*p* < 0.05 compared with K562 group treated with IM. (**B**) CD34^+^ cells from mice peripheral blood (PB) were detected by flow cytometry. *p < 0.05 versus control group. ^#^*p* < 0.05 compared with K562 group treated with IM. (**C**) CD13+ cells of BM, PB and spleen cells were detected by flow cytometry. *p < 0.05 versus control group. ^#^*p* < 0.05 compared with K562 group treated with IM. (**D**) The weight of spleens was analysis. *p < 0.05 and **p < 0.01 compared with untreated K562 group. (**E**) The weight of livers was analysis. *p < 0.05 and **p < 0.01 compared with untreated K562 group. (**F**) The expression of Stat5 and its target genes were examined by Western blot assays in bone marrow cells. *p < 0.05 and **p < 0.01 compared with untreated K562 group. (**G**) The spleens and livers were examined for H&E staining. All data was expressed as means ± SD of three independent experiments.

To examine the effect of the combination of IM and Wogonin *in vivo*, the study was performed by xenografted model. We transplanted K562 cells cultured with CM (K562 group) and CM-induced IM resistance cells (resistance group) into NOD/SCID mice by intravenous injection. 7 days later, > 1% CD13+ cells were detected in mice peripheral blood (PB), indicating that numerous human K562 cells had engrafted and proliferated in the experimental animals. Then the animals transplanted K562 cells were treated with or without IM (200 mg/kg), combination with or without Wogonin (40 mg/kg). 4 weeks later, treatment with agents alone or in combination did not result in significant weight loss compared with non-treated group (data not shown). The expression of the significant surface antigen CD34^+^ on CML was analyzed by FACS. The data indicated that CD34^+^ cells from mice BM were greatly higher levels in resistance group than in K562 group. As shown in Figure 9A, a substantial reduction in CD34^+^ cells in BM cells obtained from combination group was observed. The data of the IM-treated K562 group was 20.54 × 10^5^/ml and the combined group was 9.34 × 10^5^/ml. Similar results in CD34+ cells reduction were also found in PB (Figure 9B). In addition, there was significantly reduction in CD13^+^ cells in the combination group compared with non-treated K562 group (Figure 9C). Organs (spleen and liver) were excised from the animals and weighed. Administration with the combination restored the weight of normal spleens and livers, but the IM-treated K562 group did not restore (Figure 9D and 9E). Moreover, western blot of BM cells revealed that mice treated with combination had reduced Stat5 phosphorylation and its related anti-apoptotic proteins including Bcl-xl and Mcl-1 more significantly than single treatment group (Figure 9F). Histological evaluation of the tissues revealed marked leukemia cell infiltration of the spleen and liver in non-treated K562 group. Subsequent to treatment with Wogonin combined with IM, the histology of spleen and liver got back to normal (Figure 9G).

## DISCUSSION

The successful clinical therapy of second-generation ABL inhibitors revealed that the understanding of molecular mechanisms of IM resistance is of decisive clinical value [35, 36]. However, disease relapse and its significance for the development of outright clinical resistance is still little understood. BCR-ABL-positive LSCs persist in CML patients, despite effective inhibition of BCR-ABL after treatment with TKI [37]. Recent report showed a significantly enhanced mRNA expression of ABCB1, ABCC1 and Stat5a in patients displaying secondary IM resistance without BCR-ABL1 mutations. Here, we found that CM contributed to BCR/ABL- independent IM resistance by enhancing LSCs survival via activating Stat5/NF-κB signaling.

Although CM induced IM resistance in K562 cells was reported, few research has been undertaken to evaluate the mechanism of resistance in K562 cells subpopulation by CM-derived HS-5. In this paper, culture with CM significantly increased the proportion of CD34^+^ cells compared with RM both in the presence of IM. Moreover, the more K562 cells were eradicated by high dose of IM, the higher the proportion of CD34^+^cells in K562 was. There might be two possible reasons: (1) Culture with RM significantly reduced the survival and proliferation of CD34^+^ subpopulation in K562 cells, but CM protected them from apoptosis following IM treatment. The proportion of CD34^+^ cells in K562 was relatively increased. (2) In the presence of IM, the survival and proliferation of CD34^+^ CML cells was induced by cytokine-enriched CM, but not by RM. Thus, the proportion of CD34^+^ cells in K562 was absolutely increased. CD34+ is a stem cell marker. A report showed CD34+ subpopulation in K562 cells was much less sensitive to IM than the bulk, which supported that cancer stem cells are the mainly cause of drug resistance [34]. Our results indicated that IM alone may not be sufficient to eradicate residual CML-LSCs. Within the BM microenvironment, residual CML-LSCs might be activated when CML cells was blocked by BCR-ABL inhibitors. Thus, it is important to clarify cytokine- activated leukemia stem cell signaling pathways to develop more efficient therapies for patients with CML.

Next, we further analyzed the mechanism of CD34^+^ subpopulation survival in K562 cells and KU812 cells after IM treatment in microenvironment. The studies identified Stat5 independent of BCR/ABL as an important regulator that attenuated inhibition of IM in microenvironment. Both IL-3 and GM-CSF are similar as strong activators of JAK2/Stat5, which are critical antiapoptotic and transforming targets of BCR/ABL [14]. For example, a recent report indicated that GM-CSF can contribute to IM resistance by activating the JAK2/Stat5 pathway in CML cells [18]. Thus, we postulated that, in microenvironment, JAK2/Stat5 pathway could compensate for BCR-ABL dependent Stat5 activation when BCR-ABL was blocked by IM. Our results showed a significant increase of p-Stat5 and its target genes in CM cells with IM treatment, while no significant change without IM treatment. This discrepancy may be due to the very low proportion of CD34^+^ in K562 cellswhich was reactivated by in cytokine-enriched BM microenvironment. Moreover, CML CD34+ cells became more reliant on BCR-ABL independent Stat5 pathways when BCR-ABL was fully inhibited. Based on the established function of JAK2/Stat5 in conferring cell survival under inhibited conditions by TKI, it was extremely conceivable that p-Stat5 levels might be reactivated through cytokine-mediated JAK2 signaling pathways.

Constitutive NF-κB activity has been found in CM-induced IM resistance cells with activation of Stat5 (Figure 4B). This finding provided a supposition for a functional interaction between Stat5 and NF-κB in CM-induced IM resistance K562 cells. Tetsuya et al. found that GM-CSF as well as IL-3 activated NF-κB-dependent transcription, and Stat5 increased DNA binding activity and transactivation potential of NF-κB [38]. Similar results from other groups clarified that activated Stat5 enhanced the DNA-binding affinity of RelA on target promoter sequences [22]. Our data showed that Stat5 functionally interacted with NF-κB signal transduction pathways in the resistant cells. These are likely to include several distinct mechanisms where Stat5 inhibited the function of other transcription factors such as GR and PPARα: the direct protein-protein interaction-mediated mechanism for the former, and the yet unidentified indirect mechanism for the latter. We detected a positive association between Stat5 and the DNA binding activity of NF-κB. Data strongly indicated a direct mechanism involving as yet unidentified cytokine (s) in Stat5-dependent NF-κB activation.

A number of recent studies have indicated TNFα has been found to be frequently upregulated and is important for inflammation-induced cancer through activating NF-κB. In the present study, the results did not contradict these findings but rather indicated that constitutive NF-κB activity regulating multiple critical signaling pathways was determined in part by its interaction with activated Stat5. We further found that p-Stat5 and p-RelA also physically bound with each other in nucleus. Phosphorylated Stat3 was able to interact with p300/phosphorylated RelA efficiently, leading to RelA acetylation [39]. Here, we investgated whether Stat5 activation led to enhancement of the DNA binding activity of NF-κB though RelA acetylation. As expected, IP assay suggested that Stat5 activity inhibited RelA affinity for IkBα, which might contribute to interact with p-Stat5 and Ac-RelA in nucleus. Although knockdown of IKK did not abrogate NF-κB activity presented in the tested tumor cells, our results did not challenge the importance of IKK activity in the carcinogenic process. In fact, many of these cytokines and growth factors are encoded by NF-κB target genes that require IKK activation [40].

Our data suggested that suppressing Stat5 directly was an attractive approach to overcome resistance to BCR/ABL kinase inhibitors. However, STAT5 was a difficult drug target as it lacks an enzymatic domain. Hence, we analyzed the expression of Stat5 by Wogonin, which is now increasing evidence effective antileukemic properties and low-toxicities [41]. Cheng et al. reported that magnetic nanoparticles of Fe_3_O_4_ and Wogonin sensitized multidrug resistance K562/A02 cells to cancer therapy *in vitro* [42]. Enhancement of apoptosis and reduction of telomerase activity were mediated by Wogonin in the HL-60 leukemia cells [43]. Yang et al. reported cell cycle arrest and erythroid differentiation were induced by Wogonin in imatinib-resistant K562 cells and primary CML cells [44]. In addition, the combination of Wogonin and magnetic nanoparticles provided a promising strategy for lymphoma therapy [45]. In this study, we found that, in a safe dose, Wogonin potentiated the inhibitory effect of IM via suppressing Stat5 pathway *in vivo* or *in vitro*.

**Figure 10:**
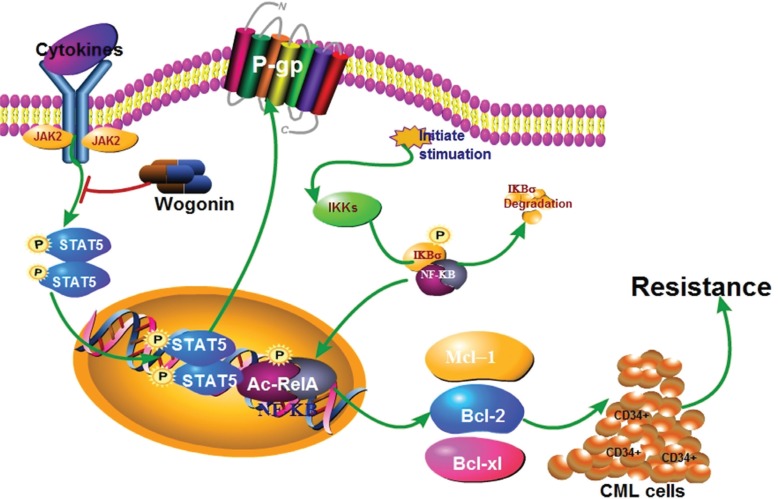
Schematic representation of the molecular mechanisms proposed in BM microenvironmental protection of CML cells from IM

As shown in Figure 10, this study addressed a reasonable mechanism that BM microenvironment protected CML-LSC cells from IM-induced cell death through BCR/ABL-independent activation of Stat5, consequently upregulated the DNA binding activity of NF-κB though binding of p-Stat5 and p-RelA, and resulted in RelA acetylation in nucleus. Further, Wogonin effectively decreased Stat5 function in BM microenvironment. Therefore, identification of Wogonin was a potent small-molecule inhibitor of Stat5, and developing it into druggable compounds might be used to increase the efficacy of anticancer drugs. Thus, further studies in human clinical trials are necessary to test the efficacy of the combination of IM and Wogonin as well as other Stat5 inhibitors during the prevention and treatment of resistant patient.

## MATERIALS AND METHODS

### Materials

Wogonin was isolated from *S. baicalensis Georgi* according to previous protocols [44]. Wogonin was of ≥ 99% or higher in all experiments, unless otherwise noted. Wogonin was dissolved in dimethyl sulfoxide (DMSO) as a stock solution (100 mM), stored at −20°C, and diluted to each of the designated concentrations in the buffer solution before each experiment. The final concentration of DMSO did not exceed 0.1%. IM was purchased from Melonepharma (Dalian, China). Primary antibodies of β-actin (1:2000), P-gp (1:500), NF-κB (1:500), p-RelA (1:500), IKKβ (1:500), IκBα (1:500) and Caspase 3 (1:500) were obtained from Santa Cruz (Santa Cruz, CA). p-Stat5 (1:1000), Stat5 (1:1000), Mcl-1 (1:1000), Bcl-2 (1:1000), BCR/ABL (1:1000), Bcl-× l (1:1000) and Lamin A (1:1000) were from Bioworld (OH, USA). RPMI-1640 (Gibico, Carlsbad, CA) and DAPI (Invitrogen, USA) were purchased. The IRDye^™^ 800 conjugated secondary antibodies were the products of Rockland Inc. (Philadelphia, PA). FITC-conjugated anti-human CD13 antibody and PE-conjugated anti-human CD34 antibody were purchased from eBioscience. Stat5 siRNA and IKKβsiRNA were from Santa Cruz Biotechnology. Stat5-luc Plasmid and NFκB-luc Plasmid were obtained from Beyotime (Nanjing, China).

### Cell culture and animals

Human CML K562 and KU812 cells was obtained from the American Type Culture Collection (ATCC). The cells were cultured in RPMI 1640 supplemented with 10% fetal bovine serum [Gibco, USA; regular medium (RM)] at 37°C in 5% CO2 in ahumidified incubator. The human stromal cell line HS-5 (obtained from the ATCC) was cultured under the same condition.

The animal study was carried out according to the regulations of the State Food and Drug Administration (SFDA) of China on Animal Care. NOD/SCID immunodeficient mice (aged 5–6 weeks) were purchased from Shanghai Slac Laboratory Animal Company Limited. The mice were raised in air-conditioned pathogen-free rooms under controlled lighting (12 h light/day) and fed with standard laboratory food and water. K562 cells (K562 group) and CM-induced IM resistance K562 cells (resistance group) at 2 × 10^6^ were injected into each mouse via tail vein. After one week, the mice inoculated with K562 were randomized into four groups (6 mice per group): (1) Untreated group as a negative control; (2) Wogonin monotherapy (40 mg/kg); (3) IM monotherapy (200 mg/kg); (4)Wogonin combined with IM. Wogonin was given intravenously and IM was administered orally. Wogonin was given once every other day and IM was given once every day. The mice were euthanized when became moribund at the fifth week, all surviving mice were euthanized. Bone marrow, peripheral blood and spleen cells were collected. The leukemia cells and CML stem cells were detected by flow cytometry after labeled with FITC-conjugated anti-human CD13 antibody (eBioscience), PE-conjugated anti-human CD34 antibody (eBioscience), respectively.

### MTT assay

The MTT assay was performed to determine the survival rate of cells incubated with the anticancer drugs (IM, Wogonin) at various concentrations. After dilution in RM or CM for 36 h, 20 μl MTT dye was added to each well and incubated for an additional 4 h. The dye was solubilized with 100 μl of DMSO, and the plates were read at 570 nm on an automated microtiter plate reader. A blank well that contained only media and drug was used as a control for all the experiments. The inhibitory ratio was calculated using the following formula: inhibitory ratio (%) = (1−the average absorbance of the treated group/the average absorbance of the control group) × 100%.

### Cell proliferation detection

To detect cell proliferation, cells were harvested after treatment and then processed with Ki67 cell proliferation Detection Kit (KeyGen Biotech, Nanjing, China) according to the manufacturer's instructions. Observation was taken under a light microscope.

### Annexin V/PI staining

Cells were harvested after treatment and stained with the Annexin V/PI Cell Apoptosis Detection Kit (KeyGen Biotech, Nanjing, China) according to the manufacturer's instructions. Data acquisition and analysis were performed with a Becton Dickinson FACS Calibur flow cytometer using Cell-Quest software at Ex./Em.-488/530 nm. The cells in early stages of apoptosis were Annexin V positive and PI negative, whereas the cells in the late stages of apoptosis were both Annexin V and PI positive.

### Western blot analysis

Cells were collected and lysed in lysis buffer (100 mM Tris-HCl, pH 6.8, 4% (m) SDS, 20% (v) glycerol, 200 mM β-mercaptoethanol, 1 mM PMSF, and 1 g/mL aprotinin) for 1 h on ice. The lysates were clarified by centrifugation (13, 000 rpm) at 4°C for 15 min for 30 min. Protein concentrations in the supernatants were measured using a bicinchoninic acid (BCA) assay kit (Pierce, Rockford, IL, USA) by a Varioskan multimode microplate spectrophotometer (Thermo Waltham, MA, USA). Equal amounts of protein were separated by 8–12% SDS-PAGE. The blots were then incubated with primary antibodies for overnight at 4°C, followed by incubation with IRDyeTM800 conjugated secondary antibody for 1 h at 37°C. Detection was performed using the Odyssey Infrared Imaging System (LI-COR Inc, Lincoln, NE, USA). All blots were stripped and reprobed with polyclonal anti-β-actin or anti-LaminA antibody to ascertain equal protein loadings.

### DAPI staining

To detect morphological evidence of apoptosis, cell nuclei wasvisualized following DNA staining with the fluorescent dye DAPI (Santa Cruz, USA) as previously reported. Briefly, K562 cells were cultured in 6-well plates and treated with the indicated concentration of drug. At the end of incubation, the cells were fixed with 4% paraformaldehyde for 20 min and incubated with DAPI (1 μg/mL) for 10 min. After washing with PBS, the nuclear morphology of cells was examined by fluorescence microscopy (Olympus, Japan) with a peak-excitation wave length of 340 nm.

### Rhodamine-123 intracellular accumulation

The efflux of chemotherapeutic drugs from tumor cells into the surrounding tissue is related to P-gp. K562 cells were harvested after treatment and 5 μg/ml rhodamine 123 (the fluorescent P-gp substrate) was added. After incubation for 30 min, cells were placed in ice water to cease the reaction, harvested and washed twice with ice-cold PBS. Subsequently, samples were analyzed by FACS Calibur (Becton-Dickinson). Excitation wavelength and emission wavelength are 488 and 530 nm, respectively.

### Immunofluorescence confocal microscopy

Treated K562 cells were harvested and seeded onto glass coverslips processed for immunofluorescence. In brief, cells were fixed with 4% paraformaldehyde (PFA) and incubated with Triton X-100. Following the incubation, the cells were blocked with PBS containing 5% BSA for 1 h, and incubated with anti-Stat5 antibody (1:50) or anti-p-RelA antibody (1:50) at 37°C for 1 h. After washing with PBS containing 0.01% Tween 80, the cells were stained with FITC-conjugated anti-rabbit or anti-mouse antibody (1:100) for 1 h. And then the coverslips were stained with DAPI for 30 min. The images were observed under confocal microscopy at 1000 × magnification (FV1000; Olympus, Tokyo, Japan).

### Electrophoretic mobility shift assays (EMSA)

Nuclear extract was isolated from K562 cells after treatment. According to the manufacturer's protocol, the experiments with a nonradioactive (biotin label) gel shift assay was performed (Beyotime Institute of Biotechnology, Haimen, China). Briefly, the NF-κB oligonucleotide probe was end labeled with biotin with terminal deoxynucleotidyl transferase. Following addition of 5 μl sample buffer, the DNA- protein complexes were resolved on a 6% non-denaturing polyacrylamide gel in a 0.5 × Tris-borate-EDTA buffer at 380 mA for 1 h and then transferred to nylon membrane. Finally, the biotin-labeled DNA was detected by chemiluminescence using the Chemiluminescent EMSA Kit (Beyotime, China) and exposed to X-ray film.

### Immunoprecipitation (IP)

Whole-cell lysates were incubated with anti-p-Stat5 antibody, anti-p-RelA antibody or anti-Ac-RelA antibody for 1 h at 4°C, and then added 20 μl of protein G/A agarose beads (Santa Cruz Biotechnology, St. Louis Park, Minnesota, US) overnight at 4°C. Beads were washed with cell lysis buffer 4 times and bound proteins were eluted with 2 × loading sample buffer. Then, samples were stored at −20°C for western blot.

### siRNA transient transfection

The transient transfection assay was carried out in 6-well plates with 3.5 × 10^4^/well K562 cells in 1 ml culture medium. Then either Stat5 siRNA or IKKβsiRNA was added into the cells with SuperFectinTM II *in vitro* siRNA transfection reagent (Pufei Biotech, ShangHai, China) according to the manufacturer's instructions.

### Soft agar colony-formation assay

K562 cells were treated with Wogonin and/or IM for 36 h. Cells were seeded in 6-well plates at 10000/well in 0.8% agar in RPMI-1640 culture medium over a 1.2% agar layer. Subsequently, plates were incubated for 30 days until the colonies were large enough to be visualized. To detect colony size and colony numbers, pictures at 40 × magnification were captured by an inverted microscope equipped with a color camera (Nikon Instruments, Inc., Lewisville, TX).

### Luciferase reporter assay

Cells were cotransfected with pNFκB-TA-luc (Beyotime, Haimen, China) with Renilla luciferase reporter (as internal control) for 24 h and then treated with or without IM for 36 h in CM. The luciferase activity of cell lysate was detected by the Dual-Luciferase Reporter kit (Beyotime, Haimen, China) according to the provided protocol. Luciferase signals were collected by DualLuciferase Assay system (Thermo Fisher Scientific, Rockford, IL).

### Statistical analysis

All results shown represent the mean ± SD from triplicate experiments performed in a parallel manner. Statistical analyses were performed by a one-way ANOVA using the SPSS 11.5 software.
